# Reasons for previous Cesarean deliveries impact a woman’s independent decision of delivery mode and the success of trial of labor after Cesarean

**DOI:** 10.1186/s12884-020-2833-2

**Published:** 2020-03-24

**Authors:** Kaname Uno, Michinori Mayama, Masato Yoshihara, Takehiko Takeda, Sho Tano, Teppei Suzuki, Yasuyuki Kishigami, Hidenori Oguchi

**Affiliations:** Department of Obstetrics, Perinatal Medical Center, TOYOTA Memorial Hospital, 1-1, Heiwa-cho, Toyota, Aichi Japan

**Keywords:** Trial of labor after cesarean (TOLAC), Elective repeat cesarean delivery (ERCD), Vaginal birth after cesarean (VBAC), Uterine rupture, Prelabor rupture of membranes (PROM), women’s independent decision, Mode of delivery

## Abstract

**Background:**

Cesarean delivery rates are increasing globally with almost half of them occurring due to a previous Cesarean delivery. A trial of labor after Cesarean (TOLAC) is considered a safe procedure, but most eligible women instead undergo Cesarean before 39 weeks of gestation. Lack of education about TOLAC is often associated with increased repeat Cesarean. To reveal the safety and feasibility of TOLAC, we conducted this observational, prospective study with women’s independent decisions. We aimed to clarify the relationship between their chosen mode of delivery and the reason for their previous Cesarean. Additionally, we have tried to identify maternal and obstetric factors associated with failed TOLAC to improve its success rate.

**Methods:**

This was a prospective, observational study of 1086 pregnant women with at least one previous Cesarean delivery. Of these, 735 women met our TOLAC criteria (Table 1), and then, could choose TOLAC or repeat Cesarean after receiving detailed explanations regarding the risks and benefits of both procedures. The primary outcomes were the number of successful TOLAC procedures and 5-min Apgar scores < 7 for the trial of labor after Cesarean group and elective Cesarean group. We collected the maternal and neonatal data including the reasons of previous Cesarean.

**Results:**

In total, 64.1% of women chose TOLAC. The success rate was 91.3%. The uterine rupture rate was 0.6%. There were no significant differences in the rate of Apgar scores at 5 min < 7 between both groups. Histories of experience of labor in previous Cesarean delivery were observed in 30 and 50% of women who chose TOLAC and repeat Cesarean, respectively (*p* < 0.05). Factors related to failed TOLAC included ≥40 weeks of gestation (odds: 5.47, 95% CI: 2.55–11.70) and prelabor rupture of membranes (PROM) (odds: 4.47, 95% CI: 2.07–9.63).

**Conclusions:**

TOLAC is a favorable delivery option for both mothers and neonates when women meet criteria and choose after receiving detailed explanations. Women who experience PROM or ≥ 40 weeks of gestation, their modes of delivery should be reconsulted.

## Background

Globally, Cesarean delivery rates have been steadily increasing [1, 2]. Approximately one-third to half of elective Cesarean are performed because of a history of Cesarean delivery [1, 3, 4]. In some cases, Cesarean delivery is an effective intervention to save maternal and infant lives, while perinatal complications including cerebral palsy rates or mortality have remained the same despite increasing incidence of Cesarean delivery [5, 6].

A trial of labor after Cesarean (TOLAC) can be a safe alternative and success rates are reported 60–80% [3, 7–11]. Successful TOLAC has many long- and short-term benefits, including reduced perinatal complications, bleeding, thrombosis, and infection, and later life, chronic pain and ileus [1, 11–13]. Despite this, the rate of TOLAC has been declining [8, 11]. The mode of delivery is strongly associated with counseling from doctors [3, 8]. In clinical practice, obstetricians tend to emphasize the major complications associated with TOLAC, in particular uterine rupture. Many women who are eligible for TOLAC undergo elective repeat Cesarean delivery (ERCD) before 39 weeks of gestation [14, 15]. Clinicians should provide more in depth information on TOLAC to allow women to make an informed decision.

To date, very few randomized trials have been performed that provide comparative data [16, 17] and the choice of TOLAC or ERCD are still controversial because of associated benefits and risks. In addition, lack of education about TOLAC is often associated with ERCD [4, 18–20] as the majority of women are unable to make an informed decision [8]. To reveal the safety and feasibility of TOLAC, we conducted this observational, prospective study of TOLAC with women’s independent decisions regarding route of delivery, following receipt of sufficient information about both TOLAC and ERCD.

## Methods

### Study design

This was a prospective, observational cohort study conducted from April 2005 to August 2017 at the Perinatal Medical Center of TOYOTA Memorial Hospital, Japan. At this hospital, there are approximately 500 deliveries annually. Most women have obstetrical risk factors like high maternal age, obesity, hypertension, gestational diabetes mellitus, twins, diseases of the thyroid gland, and history of myomectomy or Cesarean delivery. The success rate for nulliparous, vertex and singleton vaginal deliveries is approximately 85–90%.

We included pregnant women with one or two previous Cesarean delivery from the second trimester. Our criteria for TOLAC are presented in Table 1. We excluded women who failed to meet one or more inclusion criteria. Women who experienced spontaneous abortion, intra uterine fetal demise or whose fetuses had lethal congenital were excluded. We excluded women who underwent emergency Cesarean delivery for any condition that potentially threatened maternal life or incited non-reassuring fetal status before labor onset including severe preeclampsia, eclampsia, and placenta abruption. Women who experienced onset of labor before 34 weeks of gestation were also excluded. We did not exclude late preterm delivery (34–36 weeks of gestation) because they had already decided their mode of delivery.

**Table 1:** Criteria for TOLAC in this study

1) only one previous Cesarean delivery	
2) former Cesarean delivery was a low transverse Cesarean	
3) no obstetric contraindications for vaginal delivery	
3) singleton pregnancy	
4) vertex presentation	
5) no history of myomectomy	
6) in general, no medical induction of labor	
7) well informed on the risks of TOLAC	

*TOLAC* Trial of labor after Cesarean

All women who met criteria were provided with comprehensive information about the risks and benefits of both TOLAC and ERCD at the hospital’s outpatient unit. This information had detailed written contents at 32 weeks of gestation, including the fact that TOLAC is associated with a high risk of uterine rupture (around 0.5%), and that the success rate of TOLAC is around 80%. If uterine rupture occurs, the fetus and/or mother can die. On the other hand, ERCD is associated with higher risks of bleeding, infection, thrombosis, and complications during subsequent pregnancies compared to vaginal delivery. We explained the benefits of successful TOLAC including reduced risk of bleeding, infection and thrombosis, earlier discharge and improved outcomes in future pregnancies, including reduced risk of uterine rupture. A benefit of ERCD is avoiding the risk of uterine rupture during labor. Women were asked to consider both modes of delivery before providing written consent of their decision at 34 weeks of gestation. We supported the decisions of all the mothers and gave no intervention regarding their decision. We received written informed consents from all women about their decision. Women also provided written informed consent for emergency Cesarean delivery if required.

At onset of labor, all TOLAC women received an intravenous line and the fetal heart rate and uterine contractions were continuously and closely monitored. If required, an operating room was available within 20 min. We observed each women’s natural labor progression, and epidural anesthesia was provided upon patient request. As general rule, we did not induce labor to meet an expected delivery date or in expectation of fetal macrosomia until 41 6/7 weeks of gestation. For women without onset of labor by 42 0/7 weeks of gestation, we revisited the topic of delivery route and decided on induction of labor with oxytocin, waited one more week for spontaneous onset of labor, or undertook Cesarean delivery at that time. PROM was defined as rupture of membrane before any contraction. In cases of PROM, we observed the women for 24–48 h for signs of inflammation before induction of labor by oxytocin. In cases of non-reassuring fetal status with decreased amniotic fluid, aminoinfusion was performed.

We collected data about neonates, and maternal information about maternal age and pre-pregnancy body mass index (BMI), gestation, neonatal weight, Apgar score at 5 min, pH of the umbilical artery, estimated blood loss, the years and indications for previous Cesarean delivery and history of vaginal births and fertility treatments.

In women who chose ERCD, the operation date was anywhere from 37 to 39 weeks of gestation, determined at 34 weeks of gestation. In the case of spontaneous onset of labor before the operation date, they underwent emergency Cesarean delivery. We collected the maternal and neonatal data and compared it with the TOLAC data.

**Fig. 1:**
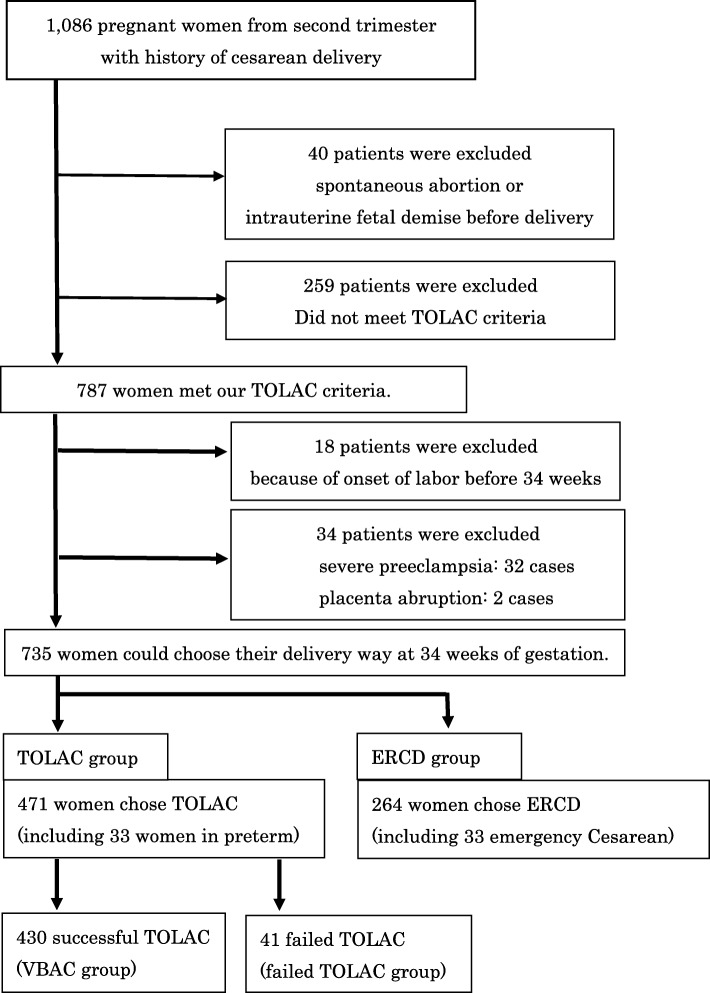
Flowchart of women included in this study

Women were classified as 1) vaginal birth after Cesarean (VBAC), 2) ERCD, and 3) failed TOLAC who chose TOLAC but who received an emergency Cesarean delivery (Fig. 1). We classified women without spontaneous labor and Cesarean delivery at more than 41 0/7 weeks of gestation as the failed TOLAC group. The TOLAC group included 1) VBAC and 3) failed TOLAC.

### Data collection

The primary outcomes were successful VBAC with informed choice and the rate of 5 min Apgar score < 7 for the TOLAC and ERCD groups. We compared age, pre-pregnancy BMI, weeks of gestation at delivery, neonatal weight, pH of the umbilical artery, and estimated hemorrhage at delivery. Success rates of TOLAC were evaluated at each week during gestation. We also examined the factors associated with failed TOLAC. Neonates admitted to neonatal intensive care unit (NICU) were monitored by neonatal doctors at outpatient unit and checked about infant deaths or long-term sequelae. Maternal information was written on templates at the time of outpatient unit by doctors and midwives. All staff shared these contents and, when necessary to prevent missing data, asked about blank data every visit. When pregnant women admitted, these information were checked again by doctors and nurses. Data associated with perinatal were stored by doctors and midwives in charge.

### Ethics

The study protocol was approved by the Ethics Committee of TOYOTA Memorial Hospital (reference No. R81). Written informed consent was obtained from all participants before participating in this study. Antenatal care was provided to all pregnant women in this study.

### Statistical analysis

Normally distributed data are reported as means ± standard deviations, and non-normally distributed data are reported as medians and minimum and maximal ranges. Categorical variables are presented numerically plus percentages. We calculated between-group differences for normally distributed data using independent sample t-tests. Non-normally distributed data were analyzed with non-parametric tests. Percentage comparisons were analyzed with Pearson’s Chi-square test. In multiple logistic regression analysis about VBAC and failed TOLAC group, we picked up the significant predictors of univariate analysis. All statistical analyses were performed with the IBM SPSS software package (version 22.0, IBM Corp., Armonk, NY, USA). The level of significance was set at *p* < 0.05.

## Results

### Overall the results

**Fig. 2:**
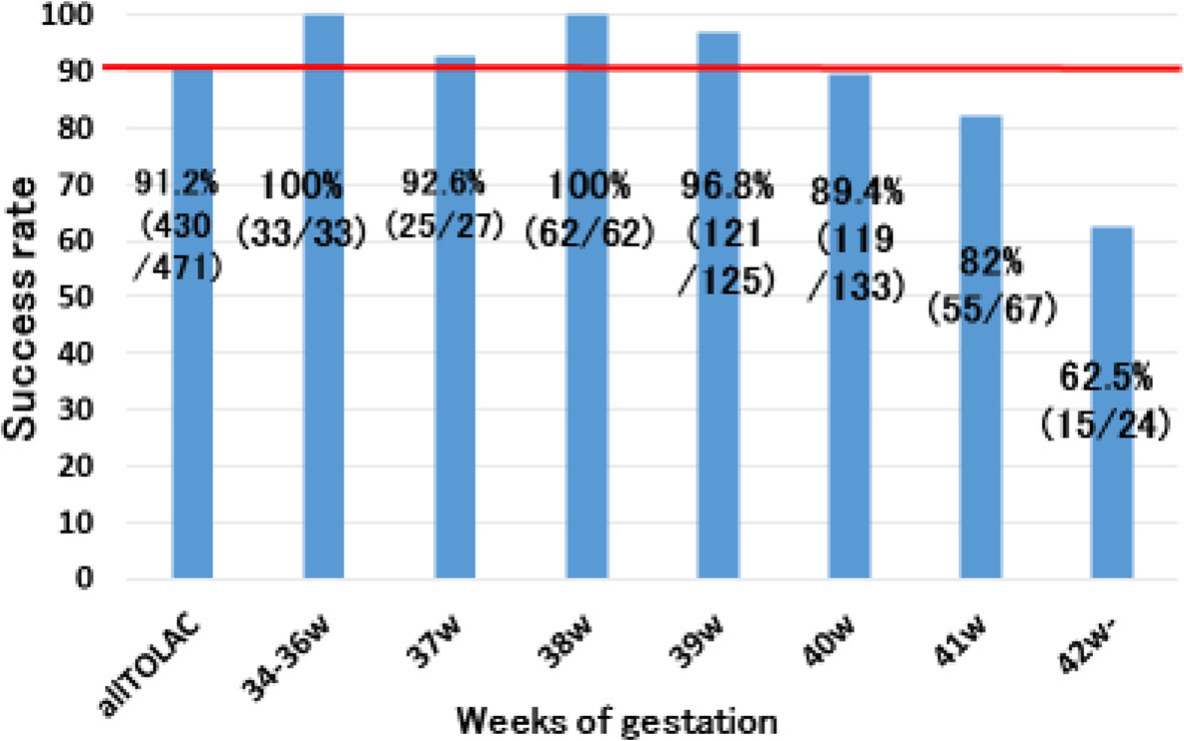
TOLAC success rates and gestational age. TOLAC success rates after 40 weeks of gestation were lower than the average (91.2%)

This study included a total of 1086 pregnant women, with at least one previous Cesarean delivery. Seven hundred thirty-five women were eligible for TOLAC and enrolled in this study (Fig. 1). Each woman made an informed decision on their mode of delivery, 471 (64.1%) women opted for TOLAC and 264 (35.9%) women ERCD. Among the 471 women who chose TOLAC, 430 (91.3%) women had a successful VBAC, 41 (8.7%) women failed TOLAC, and 3 (0.6%) women experienced uterine rupture. During this study, no maternal or neonatal deaths occurred. Among the women who underwent TOLAC, 33 experienced preterm birth. All these women had successful vaginal deliveries. The success rate of TOLAC before 40 weeks of gestation was 97.6% (241/247) (Fig. 2). After 40 weeks of gestation, success rates decreased as follows: 89.4% (119/133): 40 weeks, *P* < 0.01, 82.0% (55/67): 41 weeks, *P* < 0.01, and 62.5% (15/24): 42 weeks, *P* < 0.01 (Fig. 2). The reasons for failed TOLAC included labor arrest disorders (20/41, 48.8%), non-reassuring fetal status (13/41, 31.7%), and no onset of spontaneous labor by 41 0/7 weeks gestation (7/41, 17.1%). One patient underwent emergency Cesarean delivery due to suspected uterine rupture, however, uterine rupture was not observed. Among the women who chose ERCD, 33 (12.5%) received emergency Cesarean delivery because of early labor onset.

### TOLAC versus ERCD

Tables 2 and 3 show characteristics and outcomes of the groups. TOLAC women were significantly younger and had lower BMI than ERCD women (Table 2). The TOLAC group had significantly longer gestation than ERCD. The number of neonates whose umbilical artery pH < 7.0 were 3 only in TOLAC group. There was no significant difference in the 5-min Apgar scores < 7 between the two groups (*p* = 0.166). The most common reasons for NICU admission were preterm birth, low birth weight, and tachypnea of the newborn. The amount of bleeding at delivery in the TOLAC group was much lower than that of the ERCD group. These differences were also apparent after covariance or multiple logistic regression analyses. In TOLAC, 70.5% of TOLAC women had a previous Cesarean delivery without labor due to a non-recurrent indications, including breech presentation, placenta previa, severe preeclampsia, twins or suspected cephalopelvic disproportion. On the other hand, over 40% of ERCD women had a previous Cesarean delivery following labor, for reasons such as labor arrest disorders or non-reassuring fetal status (*p* < 0.01) (Table 2).

**Table 2:** Population characteristics of TOLAC and ERCD group

	TOLAC (*n* = 471)	ERCD (*n* = 264)	*P* value
Age	32.6 ± 4.4	33.9 ± 4.7	< 0.01
Pre-pregnancy BMI	21.8 ± 4.0	23.2 ± 5.1	0.01
Years since previous Cesarean delivery	3.0 (0–16)	3.0 (1–16)	0.24
**Previous Cesarean delivery without labor** (non-recurrent)	70.5% (332/471)	57.2% (151/264)	< 0.01
Breech presentation	185/332	78/151	< 0.01
Preeclampsia related	57/332	21/151	0.08
Placenta previa	30/332	5/151	< 0.01
Twins	24/332	12/151	0.85
Other non-recurrent	36/332	35/151	
**Previous Cesarean delivery during labor** (recurrent)	29.5% (139/471)	42.8% (113/264)	< 0.01
Arrest of labor	41/139	60/113	< 0.01
Non-reassuring fetal status	89/139	52/113	0.85
Other recurrent	9/139	1/113	

*TOLAC* Trial of labor after Cesarean, *ERCD* Elective repeat Cesarean delivery, *BMI* Body mass index

**Table 3:** Perinatal outcomes of TOLAC and ERCD group

	TOLAC (*n* = 471)	ERCD (*n* = 264)	*P* value
Neonatal weight (g)	3072 ± 444	2911 ± 413	< 0.01
pH of umbilical artery	7.28 ± 0.07	7.30 ± 0.05	< 0.01
Uterine rupture	0.64% (3/471)	0% (0/264)	0.19
Weeks of gestation	39 (34–43)	38 (34–40)	< 0.01
5-min Apgar score < 7	1.06% (5/471)	0% (0/264)	0.16
Bleeding (mL)	492 (15–4048)	894 (216–2840)	< 0.01

*TOLAC* Trial of labor after Cesarean, *ERCD* Elective repeat Cesarean delivery

### TOLAC versus failed TOLAC

The perinatal characteristics in the VBAC and failed TOLAC groups are shown in Table 4. Though the average age and years of previous Cesarean delivery did not differ, in women with successful TOLAC, BMI was significantly lower than those observed in women with failed TOLAC. The women with failed TOLAC had significantly longer gestation and higher neonatal weights. While only 9.8% of failed TOLAC women had histories of vaginal delivery, 25.1% of VBAC women had histories of vaginal delivery (*p* = 0.02). The percentage of successful TOLAC in women with prior vaginal delivery was 96.4% and there were no instances of uterine rupture. PROM occurred in 21 women (51.2%) within the failed TOLAC group. On the other hand, only 17% of the VBAC group experienced PROM, which was significantly lower than the failed TOLAC group (*p* < 0.01). Table 5 shows the results of multiple logistic regression analysis. PROM and ≥ 40 weeks of gestation were strongly associated with failed TOLAC (odds ratio 4.47 and 5.47, 95% confidence interval (CI) 2.07–9.63 and 2.55–11.70, respectively, Table 5). These factors were still associated with failed TOLAC in women, who were classified as a failed TOLAC due to no onset of spontaneous labor by 41 0/7 weeks of gestation resulting in Cesarean delivery. History of vaginal delivery was associated with successful VBAC, but was not significant (*p* = 0.19, odds 0.43, 95%CI 0.12–1.56).

**Table 4:** Perinatal characteristics of VBAC and failed TOLAC group

	VBAC *n* = 430	Failed TOLAC *n* = 41	*P* value
Age	32.6 ± 4.4	33.1 ± 4.6	0.63
Years since previous Cesarean delivery	3.0 (0–16)	3.0 (0–7)	0.56
BMI	20.9 (13.1–44.1)	23.8 (16.4–38.8)	0.03
History of vaginal delivery	25.1% (108/430)	9.8% (4/41)	0.02
Infertility treatment	10.9% (47/430)	22.0% (9/41)	0.06
No experience of labor in former Cesarean (non-recurrent)	71.4% (307/430)	61.0% (25/41)	0.16
Weeks of gestation	39 (34–42)	41 (37–43)	< 0.01
Neonatal weight (g)	3013 ± 483	3257 ± 507	0.04
Induction or augmentation of labor	19.5% (84/430)	21.9% (9/41)	0.71
PROM before onset of labor	17.2% (74/430)	51.2% (21/41)	< 0.01

*VBAC* Vaginal birth after Cesarean, *TOLAC* Trial of labor after Cesarean, *BMI* Body mass index, *PROM* Prelabor rupture of membranes

**Table 5:** Multiple logistic regression analysis of VBAC and failed TOLAC group

	*P* value	Odds ratio	95% Cl
BMI	0.03	1.09	1.01–1.18
40 weeks of gestation or more	< 0.01	5.47	2.55–11.70
PROM	< 0.01	4.47	2.07–9.63

*VBAC* Vaginal birth after Cesarean, *TOLAC* Trial of labor after Cesarean, *BMI* Body mass index, *CI* Confidence interval, *PROM* Prelabor rupture of membranes

### Brief summary of uterine rupture cases

The three women who experienced uterine rupture had no history of vaginal delivery. One woman was diagnosed with uterine rupture due to decreased variability in fetal heart tracing, experienced concomitant abdominal pain and vaginal bleeding. The other two had unsuccessful vacuum or forceps extractions and experienced intraoperative uterine ruptures. These women’s brief clinical course summaries were written in supplement file. All three neonates were admitted to the NICU with breathing disorders and monitored closely. There were no intellectual or physical disabilities observed at 1 year of age.

## Discussion

In this study, 91.3% of TOLAC were successful with no significant differences in Apgar scores in both the TOLAC and ERCD group. TOLAC is considered a reasonable means of delivery not only for mothers but also neonates. Firstly, the high success rate observed may be a direct result of the criteria for TOLAC candidacy, as shown in Table 1. TOLAC is known to increase the risk of uterine rupture. The inclusion and exclusion criteria for TOLAC in this study were discussed according to previous studies to minimize the risk of uterine rupture [4, 7, 21]. Based on this study method, the success rates were favorable. Some studies state that pregnant women with short interval since the previous Cesarean delivery or assessment of hysterotomy scar thinning should not undergo TOLAC [22]. In this study, though we did not put these into the criteria, the rate of uterine rupture did not increase compared with previous studies [3, 7, 12]. Sonographic assessment of hysterotomy scar is difficult and poor accuracy especially at second and third trimester [23].

Secondly, we believe that women’s choices are important factors for determining the success of TOLAC. That is because women who have not previously experienced labor, which means a non-recurrent indication, like breech presentation and placenta previa, tended to choose TOLAC. On the other hand, women who had emergency Cesarean delivery because of labor arrest disorders or fetal status issues tended to choose ERCD (*p* < 0.01). Women’s decision making is very important and these differences may have impacted the success rate, partly because unsuitable candidates may be excluded [4, 8, 24–27].

Additionally, our study has lower rates of epidural analgesia and reduced augmentation compared to previous studies where general management and characteristics of women were similar. Previous studies report TOLAC success rates of around 80% and rates of epidural and augmentation around 50% [28, 29]. Meanwhile, in this study epidural usage and augmentation rates were only 10 and 20% respectively. Although there is no significant difference in the percentage of women who received epidural analgesia or induction of labor, we believe this is a potential reason for the high VBAC rates observed in this study. Further studies should investigate if lower rate of epidural and augmentation play a role in the success of VBAC. In particular, women with history of vaginal delivery have higher chance of successful VBAC compared to women with no history of vaginal delivery (96.4%). When labor spontaneously occurred before 40 weeks of gestation, the success rate was favorable (97.6%).

Our results suggested that if women receive sufficient education and support, they tended to choose TOLAC as their desirable mode of delivery. A previous report found that over half of women choose planned vaginal delivery if they received comprehensive information [4, 18]. Obstetricians are responsible for providing information on all delivery options so women can make an informed decision. Women who choose TOLAC appear to be good candidates for successful VBAC.

In this study, there were no infant deaths or long-term sequelae among all 471 TOLAC attempts. Apgar scores < 7 at 5 min did not significantly differ between the two groups. In recent years, some reports suggest that ERCD should be performed no earlier than 39 weeks of gestation because of the risk of increased respiratory outcomes [14, 30]. Clinically, it is difficult to decide the date of Cesarean delivery around 39 to 40 weeks of gestation, as the emergency Cesarean delivery rate rises due to onset of labor before operation date. Women who opt for TOLAC could enjoy a longer gestation, which can be advantageous for neonates with regard to short- and long-term complications, including decreased mortality, morbidity and respiratory complications, and improved neurological, respiratory and cardiovascular outcomes [14, 30–32]. We would recommend that mothers decide the date of Cesarean delivery following 39 weeks of gestation and, if spontaneous labor occurs before the planned date, attempt TOLAC instead of emergency Cesarean delivery.

PROM and ≥ 40 weeks of gestation were significant risks for failed TOLAC (Table 4). Half of failed TOLAC cases experienced PROM. The odds ratio for women with PROM to have a failed TOLAC was 4.47 (Table 5). There are limited studies into the impact of PROM on successful TOLAC, two previous studies found PROM was associated with positive outcomes [33, 34]. This study found PROM was associated with increased risk of failed TOLAC (OR: 4.47, *p* < 0.01). These differences might be because of the definition of PROM and the timing of induction after PROM. Our PROM definition was rupture of membrane before any contraction. Therefore, the timing of PROM might be important. Further studies are needed to reveal weather PROM is negative or positive factor for TOLAC.

Almost all studies reveal the previous vaginal birth was found to be a strong predictor [2, 3, 9]. In our study, 96.4% of women with history of vaginal delivery had successful TOLAC. However, in multiple regression model, this was not a significant predictor. That might be because of relatively small number of failed TOLAC (*n* = 41). Reduced neonatal complications at over 39 weeks of gestation [14, 30] and a reduction in the success rate of TOLAC suggest both TOLAC and ERCD are viable options post due date. Obstetricians should perhaps be “encouraged” to have ongoing discussions with women about their mode of delivery after the due date. Ongoing discussions throughout pregnancy are important for preventing complications.

A limitation to this study was its prospective design that was dependent on women independent choices, without randomization or blinding. Additionally, only three women experienced uterine rupture, which is too small to comprehensively evaluate this complication.

## Conclusion

The success rate for TOLAC was > 90% in our institution and TOLAC is considered to be a reasonable option not only for mothers but also for the neonates.

## Supplementary information

**Additional file 1.**

**Additional file 2.**

**Additional file 3.**

**Additional file 4.**

## Data Availability

The datasets used and/or analyzed during the current study are available from the corresponding author on reasonable request.

## Abbreviations

BMI: Body mass index
ERCD: Elective repeat Cesarean delivery
PROM: Prelabor rupture of membranes
TOLAC: Trial of labor after Cesarean
VBAC: Vaginal birth after Cesarean
